# Modification of Intestinal Microbiota and Its Consequences for Innate Immune Response in the Pathogenesis of Campylobacteriosis

**DOI:** 10.1155/2013/526860

**Published:** 2013-11-14

**Authors:** Wycliffe Omurwa Masanta, Markus M. Heimesaat, Stefan Bereswill, Abdul Malik Tareen, Raimond Lugert, Uwe Groß, Andreas E. Zautner

**Affiliations:** ^1^UMG-Labor, Institut für Medizinische Mikrobiologie, Universitätsmedizin Göttingen, Kreuzbergring 57, 37075 Göttingen, Germany; ^2^UMG-Labor, Institut für Klinische Chemie/Zentrallabor, Universitätsmedizin Göttingen, 37075 Göttingen, Germany; ^3^Institut für Mikrobiologie und Hygiene, Charité—Universitätsmedizin Berlin, 12203 Berlin, Germany

## Abstract

*Campylobacter jejuni* is the leading cause of bacterial food-borne gastroenteritis in the world, and thus one of the most important public health concerns. The initial stage in its pathogenesis after ingestion is to overcome colonization resistance that is maintained by the human intestinal microbiota. But how it overcomes colonization resistance is unknown. Recently developed humanized gnotobiotic mouse models have provided deeper insights into this initial stage and host's immune response. These studies have found that a fat-rich diet modifies the composition of the conventional intestinal microbiota by increasing the Firmicutes and Proteobacteria loads while reducing the Actinobacteria and Bacteroidetes loads creating an imbalance that exposes the intestinal epithelial cells to adherence. Upon adherence, deoxycholic acid stimulates *C. jejuni* to synthesize Campylobacter invasion antigens, which invade the epithelial cells. In response, NF-**κ**B triggers the maturation of dendritic cells. Chemokines produced by the activated dendritic cells initiate the clearance of *C. jejuni* cells by inducing the actions of neutrophils, B-lymphocytes, and various subsets of T-cells. This immune response causes inflammation. This review focuses on the progress that has been made on understanding the relationship between intestinal microbiota shift, establishment of *C. jejuni* infection, and consequent immune response.

## 1. Introduction

*Campylobacter jejuni* is a Gram-negative, spiral-shaped, nonspore forming, and highly motile bacterium that grows optimally under microaerophilic conditions at 42°C. The genome of *C. jejuni* is roughly 1.6 to 1.7 Mbp with a G+C ratio of approximately 31% [1]. *C. jejuni* has a wide spectrum of hosts ranging from wild birds, chicken, and turkey to mammals such as cattle, swine, sheep, and humans and can also be found in milk, meat, and stock water.

*C. jejuni* is a highly prevalent commensal bacterium in all its hosts except for humans causing a bacterial food-borne illness known as campylobacteriosis. Campylobacteriosis has been found to be a usually self-limiting disease that is characterized by symptoms such as fever, abdominal cramps, bloody diarrhea, dizziness, and myalgia. However, in rare cases campylobacteriosis may lead to postinfectious complications like the Guillain-Barré syndrome (including the most severe form: the Miller-Fisher syndrome), reactive arthritis (Reiter's syndrome), postinfectious irritable bowel syndrome, and potentially immunoproliferative small intestinal disease [2, 3]. Epidemiological studies have shown that the bacterium infects all age groups of different social-economic regions all over the world, resulting in characteristic diarrhea. In developed countries, the disease has a prevalence peak in young adults and is characterized by bloody diarrhea with mucus. On the other hand, in developing countries the disease mainly affects children below the age of five manifesting as watery diarrhea leading to exsiccosis and electrolyte loss [4]. The incidence of *C. jejuni* infection varies throughout the world but remains to be a major cause of diarrheal disease in both developed and developing countries. The major sources of human infections have been found to be consumption of cross-contaminated food, contaminated milk, milk products and water, consumption of undercooked chicken, pig, and beef as well as contact with a mammalian host (e.g., pets), wild-birds, chicken, and bird droppings [2].

During the course of infection the bacterium has to pass several obstacles and to survive in different environments. After ingestion of at least 500 viable bacteria [5], *C. jejuni* has to establish in the intestinal milieu, which is influential by host antibacterial mechanisms like secretion of bile acids into the intestines and the intestinal microbiota.

It is hypothesized that the prevailing conditions (individually or collectively) within the human digestive system stimulate *C. jejuni* to synthesize virulence-associated factors that are involved in the development of campylobacteriosis [6, 7]. To support this hypothesis, studies on the mechanisms facilitating colonization and survival of *C. jejuni* in the human gut as well as on adherence, entry, endurance, and replication of the pathogen in human epithelial cells have received a lot of attention in the past.

Nowadays, distinct factors involved in the disruption of physiological colonization resistance maintained by commensal intestinal bacteria [8], are of increasing interest.

## 2. Intestinal Microbiota and Colonization Resistance

### 2.1. Intestinal Microbiota

The human digestive tract houses a plethora of commensal microbial, fungal, and protozoan species as well as viruses that are collectively referred to as intestinal “microbiota”. The intestinal microbiota is composed of bacteria from different phyla, bacteriophages, and a single phylum of archaea, yeast, and filamentous fungi [9]. The different phyla of bacteria in the microbiota are consistent from childhood to adulthood but the species distribution is not constant due to various factors. Between the delivery time of a child and an age of 5 years, the intestinal microbiota is determined by mode of delivery, breast feeding and other types of feeding, gestational age, infant hospitalization, and antibiotic treatment(s) [10, 11]. During this stage of life, bacteria of the following genera are dominant: *Bifidobacterium*, *Bacteroides*, *Lactobacillus*, *Escherichia*, *Klebsiella*, and *Clostridium*. In adult age and in children above 6 years, the composition of intestinal microbiota is complex with bacterial genera varying considerably among individuals due to age, genetics, health status, geographical location, stress, antibiotic exposure, and diet [10, 12]. In spite of the existence of various factors that influence the composition of bacterial genera in the intestines among various adults, the following bacteria phyla, namely, Bacteroidetes, Firmicutes, Proteobacteria, Actinobacteria, Fusobacteria, Verrucomicrobia, and Tenericutes, have been found to make up the adult intestinal microbiota with Bacteroidetes and Firmicutes being more dominant as compared to Proteobacteria, Actinobacteria, Fusobacteria, Verrucomicrobia, and Tenericutes [9, 13]. In addition to the bacterial species differences existing in microbiota of children ≤5 years, children >6 years, and adults, the bacterial loads in distinct regions of the intestinal tract vary considerably. For example, stomach and duodenum harbor a rather low jejunum and ileum a relatively intermediate and the distal colon by far the highest bacterial load within the gastrointestinal tract [14]. Importantly, the human host benefits from the intestinal microbiota by (i) fermentation of indigestible complex carbohydrates to absorbable short-chain fatty acids (SCFAs), (ii) detoxification of potentially harmful substances such as bile acids and bilirubin, (iii) providing an important layer of defense against invasion by pathogenic microorganisms also known as colonization resistance, and (iv) playing an important role in the proper development of immune system (see [15, 16] for detailed reviews).

### 2.2. Colonization Resistance

Colonization resistance is a physiological phenomenon exerted by the commensal intestinal microbiota, which deter pathogens from causing infections. In 1950 Bohnhoff and coworkers observed that treating mice with antibiotics prior to *Salmonella enterica* infection resulted in reduction of pathogen loads required to cause *S. enterica* associated infection [17]. Since then several studies have been carried out to understand the underlying mechanisms of colonization resistance in humans. So far, murine *in vivo* studies revealed that intestinal microbiota accomplish colonization resistance by (i) blocking pathogens from attaching to their target sites [18], (ii) depleting nutrients hence preventing pathogens from colonizing the intestines, and (iii) SCFAs and particularly bacteriocins produced as a result of intestinal microbiota metabolic activities, which prevent pathogenic bacteria from expressing distinct virulence genes. For example, butyrate has been shown to prevent *S. enterica* from expressing its type III secretion system (T3SS) and thus weakens its ability to invade epithelial cells [19]. Further, (iv) organic acids generated by intestinal microbiota metabolic activities alter intraluminal pH to levels that cannot support growth and replication of pathogens such as *Salmonella* spp. and *Escherichia coli* O157 [20], and (v) intestinal microbiota utilize most oxygen available in the gut leading to the creation of an anaerobic and capnophilic environment that affects the ability of some enteric pathogens to colonize the gut [21]. However, the role of *archaea*, yeast, filamentous fungi, protozoan, and helminthic parasites as well as viruses including bacteriophages in colonization resistance is not fully understood so far.

## 3. Products of Intestinal Microbiota Metabolism Affecting **C. jejuni**

The colonic microbiota utilizes dietary compounds, which remain undigested and/or unabsorbed in the small intestine as growth and replication substrates. This process leads to formation of a number of metabolic products that (i) help microbiotic bacteria species to grow and replicate, (ii) are beneficial but, (iii) have also been found to be harmful to the human host (see [14, 22] for detailed reviews). Interestingly, metabolic end products derived from the intestinal microbiota aid *C. jejuni*, for instance, to colonize the human gut and invasion of epithelia cells in the following ways.

*(a) Short Chain Fatty Acids as a Carbon Source*. Like other undigested food compounds, complex dietary carbohydrates and proteins that remain undigested in the human small intestinal lumen are transported to the large intestinal tract for further digestion and absorption. The large intestine of a healthy human adult mainly harbors bacteria of the following phyla: Bacteroidetes, Firmicutes, Actinobacteria, Verrucomicrobia, and Proteobacteria. These bacteria are capable of anaerobically breaking polysaccharides, oligosaccharides, proteins, peptides, and glycoproteins down to form SCFAs, principally, acetate, formate, lactate, butyrate, propionate, valerate, caproate, and succinate [23]. However, the SCFA yield depends on the availability of carbon sources within the large intestine. In conditions of higher carbon availability (“excess”), the major SCFAs generated are acetate and formate, whereas in situations of low carbon availability, lactate and acetate are the major SCFA generated [24]. Interestingly, previous reports showed that *C. jejuni* utilizes acetate and lactate as carbon sources [25, 26]. Furthermore, lactate and acetate consequently contribute to colonization of *C. jejuni* in human gut.

*(b) Signaling the Synthesis of Virulence-Associated Factors*. *C. jejuni* has been shown to utilize the end products of the gut microbiotical biotransformation of bile salts and it has been demonstrated that bile salts function as induction signals for synthesis and secretion of some *C. jejuni* virulence-associated factors via the flagellar type III homologue secretion system [6]. 

Bile salts are initially synthesized in the human liver. Cholic acid (CA; 3*α*, 7*α*, 12*α*-trihydroxy-5*β*-cholan-24-oic acid) and chenodesoxycholic acid (CDCA; 3*α*, 7*α*-dihydroxy-5*β*-cholan-24-oic acid) are the only bile salts that are synthesized by hepatocytes. Hence, they are named primary bile salts. Upon synthesis, these bile salts circulate in a process known as enterohepatic circulation, in which they are secreted into the gallbladder, then into the duodenum, from which they reach jejunum and ileum as well as in the large intestine. There they are reabsorbed by active transport into the blood system and finally transported to the liver for resynthesis and subsequently released into the gallbladder (again) [27, 28]. The unabsorbed bile salts are transported along with other undigested products to the large intestine. There reside anaerobic gut bacteria of the genera *Lactobacillus*, *Eubacterium*, *Bacteroides*, *Clostridium*, and *Escherichia*, which express different bile salt hydrolases. These transform the unabsorbed bile salts by deconjugation and oxidation of hydroxyl-groups at C-3, C-7, and C-12, as well as 7*α*/*β*-dehydroxylation into their respective unconjugated free bile acids commonly known as secondary bile acids [22]. Ridley et al. used the term biotransformation to describe deconjugation, oxidation of hydroxyl-groups at C-3, C-7 and C-12, and 7*α*/*β*-dehydroxylation of unabsorbed primary bile salts [29]. During biotransformation, cholic acid (CA; 3*α*, 7*α*, 12*α*-trihydroxy-5*β*-cholan-24-oic acid) is transformed into deoxycholic acid (DOC; 3*α*, 12*α*-dihydroxy-5*β*-cholan-24-oic acid) while chenodeoxycholic acid (CDCA; 3*α*, 7*α*-dihydroxy-5*β*-cholan-24-oic acid) is transformed into lithocholic acid (LCA; 3*α*-hydroxy-5*β*-cholan-24-oic acid) [29]. Human gut bacteria transform unabsorbed primary bile salts to enhance their colonization of the human gut by reducing the toxicity of the primary bile salts, to obtain carbon, nitrogen, and sulfur and to generate substances for cellular biosynthetic reactions and electron transport phosphorylation [30–33].

Interestingly, various studies have found secondary bile salts in particular DOC to induce the expression of genes that encode for so-called *Campylobacter* invasion antigens (Cia) that have been shown to play a pivotal role in the invasion of epithelial cells and survival within the epithelial cells [6, 7, 34]. A microarray study carried out by Malik-Kale and coworkers in 2008 revealed that a total of 202 genes in the *C. jejuni* strain F38011 were affected when *C. jejuni* was cultured in media containing DOC [6]; in particular, 150 genes were up-, whereas 48 were downregulated [6]. 

These results amend earlier studies, which revealed that *C. jejuni* synthesizes at least 14 additional proteins upon cocultivation with cultured mammalian cells that are not thermotolerance-associated [35, 36]. Furthermore, these findings underline previous experiments, which revealed that *C. jejuni* synthesizes a number of proteins during growth in rabbit ileal loops that are not expressed under standard laboratory (*in vitro*) conditions [37]. The findings derived from these three studies might support results obtained in a recent study showing that eight proteins were excreted into the medium when *C. jejuni* was cultured with INT 407 cells or in INT 407-conditioned medium [38]. The Cia proteins are secreted via a flagellar type III homologue secretion system [39]. The role of lithocholic acid in pathogenesis of *C. jejuni* is unknown until now.

*(c) Serving as Electron Acceptors for the C. jejuni Highly Branched Electron Transport Chains*. As mentioned above, the bile salt metabolism of intestinal microbiota yields fumarate as one of its end products. *C. jejuni* has been shown to express a methylmenaquinol fumarate reductase (Mfr) located in the periplasm, which utilizes, as the name suggests, methylmenaquinol (mMKH2, *E*m,7 −124 mV) in fumarate reduction [40]. This enzyme enables *C. jejuni* to use fumarate as an electron acceptor in oxygen limited conditions [41]. This and other oxygen-independent electron-transport chains, namely, nitrate, nitrite, and trimethylamine-*N*-oxide (TMAO) as well as dimethyl sulfoxide (DMSO), are believed to contribute to *C. jejuni's* unique ability to colonize different environments [42].

## 4. Shifts in Intestinal Microbiota Composition and **C. jejuni ** Enteritis

Reduced bacterial phyla ratio numbers in the intestinal microbiota of a healthy individual are accompanied by an abrogated physiological colonization resistance facilitating infection with pathogens. Furthermore, diseases such as type 1 diabetes [43], type 2 diabetes [44], obesity [45], inflammatory bowel disease (including Crohn's disease and ulcerative colitis) [46], irritable bowel syndrome [47], and celiac disease [48], among others, have been shown to be associated with shifted microbiota composition.  Table 1 provides a summary of altered bacteria phyla ratios in the respective diseases and disorders.

As aforementioned, several factors including genetics, geographical location, diet, and antibiotic exposure, among others, can affect intestinal colonization resistance. Most recent studies have unequivocally revealed that the diet is a primary factor causing disruption of the intestinal microbiota-mediated colonization resistance. Recently, a study was carried out to compare the intestinal microbiota of children aged 1–6 in a rural African village situated in an environment that is still comparable to that of Neolithic subsistence farmers with the intestinal microbiota of western European children of the same age, consuming typical western diet. The study revealed that Actinobacteria and Bacteroidetes were abundant in the children living in the rural African village, whereas Firmicutes and Proteobacteria were abundant in the European children [55]. Furthermore, the relative abundance of Firmicutes in the European children cohort was twofold higher as compared to the children population in the rural African village. *Prevotella*, *Xylanibacter*, and *Treponema* were exclusively present only in African children, whereas *Enterobacteriaceae* that are commonly abundant in European children were not detected in the rural African children. These findings are well in line with an earlier study by Turnbaugh and coworkers, detecting increased bacterial abundance (levels) of two classes of the phylum Firmicutes: (i) class Erysipelotrichi (*Clostridium innocuum*, *Eubacterium dolichum*, and *Catenibacterium mitsuokai*) and (ii) class Bacilli (mainly *Enterococcus* sp.). In addition, a drastic reduction in bacteria levels of the phylum Bacteroidetes was assessed when humanized C57BL/6J mice, initially kept on a standard low-fat-plant-polysaccharide-rich diet were switched to a high-fat, high-sugar western diet [56]. For a detailed review on how diet disrupts the intestinal microbiota, refer to [57].

Interestingly, a diet-induced intestinal microbiota shift was recently linked to susceptibility for *C. jejuni* infection [58]. Gnotobiotic mice fed a human western-style diet for six weeks displayed a microbiota composition more comparable to humans than to conventional mice controls. In turn, these “humanized” mice were susceptible to *C. jejuni* infection, whereas gnotobiotic mice that were fed standard murine chow established a murine microbiota composition accounting for colonization resistance against the pathogen-like conventionally colonized controls [58]. A quantitative survey of the bacterial species abundant in the colonic lumen of the mice on human western-style diet, standard murine diet, and gnotobiotic mice reconstituted with a complete human microbiota revealed that western-style diet fed mice harbored higher *E. coli* and *Clostridium/Eubacterium* spp. counts and lower *Enterococcus* and *Lactobacillus* spp. loads as compared to standard murine diet fed mice. This study may support the observation that individuals consuming western diet are more prone to *C. jejuni* infections as compared to those on a rather low-fat plant polysaccharide-rich diet [55]. The role of commensal *E. coli* in abrogating intestinal colonization resistance against *C. jejuni* has been previously shown by Haag et al. [59]. As long as the intestinal *E. coli* loads of adult mice harboring a conventional microbiota were artificially elevated by feeding a commensal murine *E. coli* strain via the drinking water, mice could be stably infected by *C. jejuni* at high loads, whereas conventional control animals could not. Current and future studies will further dissect the impact of distinct bacterial species within the triangle relationship of commensal bacteria, intestinal pathogens, and innate host responses [8, 59].

## 5. Stage Model for the Pathogenesis of **C. jejuni**

After overcoming the intestinal microbiota barrier located on and within the intestinal mucin layer, *C. jejuni* has to pass the mucosal epithelial barrier. Although not all *C. jejuni* virulence-associated factors are known up to now, the present findings have led to the construction of a stage model that attempts to explain to role of the associated virulence determinants involved in the pathogenesis of *C. jejuni* infection at this point. These stages are (1) motility and chemotaxis, (2) adherence to, translocation, and invasion of intestinal epithelial cells (IECs), (3) toxin production, (4) survival in the epithelial cells, and (5) immune response and inflammation of the intestinal epithelium [7, 60].

The expression of specific gene sets could be attributed to these different stages: Firstly, the *fla*A and *fla*B genes (flagellin A & B that assemble flagella) and the regulatory gene *che*Y (chemotaxis regulatory protein) are expressed. Flagella are responsible for motility while *cheY* plays a role in chemotaxis [61–63]. Secondly, the gene products of *peb*1A (periplasmic bifunctional adhesin/ABC transporter aspartate/glutamate-binding protein), *cad*F (cadherin F—outer membrane fibronectin-binding protein), *jlp*A (surface lipoprotein), *pfl*A (paralysed flagellum protein), *cia*B, *cia*C, *cia*I (*Campylobacter* invasion antigens B, C & I), and a cluster of lipooligosaccharide (LOS) biosynthesis genes as well as pseudaminic acid and legionaminic acid biosynthesis genes are synthesized. They play a distinct role in adhesion and invasion [7, 38, 64–69].

Thirdly, the gene products of *cdt*A, *cdt*B, and *cdt*C assemble the trimeric cytolethal distending toxin (CDT). CDT contributes to the cytopathic effect associated with *C. jejuni* infection [70]. Fourthly, the genes *chu*ABCD, *ceu*BCDE, *cfr*A, *fhu*ABD, *feo*B, *tonB*-*exbB*-*exbD*, *cft, and per*R essentially involved in iron homeostasis are expressed [29, 60, 71–75]. It should be noted, however, that in a subgroup of *C. jejuni* isolates, that exhibit an extended amino acid metabolism [76], the ferric uptake receptor *cfrA* is replaced by a protein of unknown function and a second iron uptake transport system encoded by the genes *cj0173c*-*cj0182* is missing critical components, such as *cj0178* and *tonB3*, for instance [77, 78]. Fifthly, gene *sod*B (superoxide dismutase) and the genes *kat*A (catalase) and *ahp*C (alkyl hydroperoxide reductase) contributing to superoxide stress defense and peroxide stress defense, respectively, are expressed [79–81]. Finally, the genes *gro*ES (cochaperonin), groEL (chaperone), *dna*J (chaperone), *dna*K (chaperone), and *clp*B (ATP-dependent Clp protease ATP-binding subunit) that play a role in thermotolerance are expressed [36, 82–84]. It has been shown that *C. jejuni* changes its respiration mode to fumarate respiration and undergoes metabolic downshift in order to survive in mammalian cells [33].

## 6. *C. jejuni* Induced Innate and Adaptive Immune Responses in the Gastrointestinal Tract 

The human gastrointestinal tract is endowed with a complex immune system that is made up of cells, tissues, and immune effector molecules constantly and efficiently communicating with each other in order to eliminate invading microbial pathogens. As discussed above, a shift in the intestinal microbiota composition leaves the intestinal epithelial cells (IECs) vulnerable to invasion by *C. jejuni* consequently activating both the innate and adaptive immune system [85]. The major site of bacteria mediated inflammation and tissue destruction is located in the colon, but there is evidence showing that the mucosal immune responses include the ileum because *C. jejuni* is transported across the epithelial barrier via ileal M-cells [86].

Upon adhesion and invasion of the colonic epithelium, *C. jejuni* induces IL-8 secretion, which to some extent is initiated by the cytolethal distending toxin (CDT) [87]. The IL-8 response is the initial trigger for acute mucosal inflammation characterized by neutrophil infiltration and macrophage activation, as well as proliferation of T- and B-lymphocytes. Besides these immune cell aggregates immunopathological features observed in colonic biopsies of infected patients include apoptotic crypt drop-outs, microabscesses, and focal ulcerations [88, 89]. The corresponding IgA and IgG antibodies produced by mature B-cells against *C. jejuni* are considered to contribute to long-term protection against reinfection, but they might be detrimental when cross-reacting with gangliosides in neurons which in turn results in neurological sequelae such as GBS in about 1 out of 900 infected patients [90].

*C. jejuni* induced innate immune responses are initiated by binding of bacterial cell wall compounds to nucleotide-binding oligomerization domain (NOD) or to Toll-like receptors (TLRs). At the cellular level adhering and invading *C. jejuni* are detected by Toll-like-receptor 4 and NOD1/CARD4, respectively. Innate immune signaling results in the activation of nuclear factor kappa B (NF-*κ*B) [91]. Further, NF-*κ*B stimulates production of various cytokines, which in turn mediate maturation of dendritic cells (DCs) into antigen presenting cells (APCs) and shape subsequent B- and T-cell responses (Table 2 provides a summary of these cytokines), whereas the activation of M*φ*-phagocytic cells, monocytes, and neutrophils finally leads to direct elimination of *C. jejuni* [92–94]. Within this scenario, human *β*-defensins have been shown to attack *C. jejuni* by rupturing the cell membrane [95]. While the role of B-cells and the role of the antibody responses in GBS on one hand and protection against disease on the other is well understood [90], innate immunity of campylobacteriosis and corresponding T-cell responses await further investigation and are thus in the focus of current and ongoing research. The lipooligosaccharide (LOS) of *C. jejuni* and the corresponding innate receptor toll-like-receptor (TLR) 4 were shown to be of pivotal importance for induction and progression of experimental [96] and human campylobacteriosis [97]. Notably, the LOS structure of *C. jejuni* is highly variable and it was shown that decoration of LOS with sialic acid residues aggravates the inflammatory response and the outcome of human campylobacteriosis [97]. The elevated pathogenic potential of *C. jejuni* strains with sialylated LOS (subtypes A, B, and C) is explained by the increased binding of sialylated LOS to TLR-4 [98, 99]. The resulting hyper-activation of TLR-4 signaling further drives ulcerative enteritis, bloody diarrhea, fever, and postinfectious sequelae including GBS [97]. The important role of TLR-4 signaling in *C. jejuni* immunopathology was confirmed in murine models of disease [87, 90] and corresponding results demonstrate that LOS is a key virulence factor of *C. jejuni*. Besides TLR-4, other TLRs such as TLR-2 and TLR-5 play only a minor role in *C. jejuni* enteritis [90] indicating that *C. jejuni* flagellin and lipoproteins are not crucial for activation of the immune system [100].

The *C. jejuni* induced adaptive T-cell responses in the intestines have been recently dissected in an elegant study using *ex vivo* infected explants of human colon tissue [69]. In these biopsies *C. jejuni* reached the subepithelial compartments following adhesion and invasion of IECs. The direct contact of the invading bacteria with neutrophils, macrophages, and dendritic cells initiated Th1 and responses characterized by the release of IFN-*γ*, IL-1*β*, IL-12, and IL-6 [69]. Increased IL-12 and IL-23 levels in these artificially infected biopsies were indicative of DC activation and maturation, which is subsequently driving the response of distinct Th17 cell subsets. Elevated numbers of Th17, Th1, and Th17/Th1 double-positive cells in the *ex vivo* infected biopsies were well in line with increased concentrations of IL-22 and IL-17 which finally orchestrate the eradication of the pathogen by induction of innate responses and defensin production in the epithelium [69].

## 7. Recent Advances in Developing Murine Models Mimicking Human Campylobacteriosis

Although human campylobacteriosis is of global importance, efforts geared towards understanding the underlying molecular mechanisms of *C. jejuni* infection and associated immune responses have not yielded profound insights due to the lack of appropriate *in vivo* models. Chicken, newborn piglets, weanling ferrets, gnotobiotic canine pups, primates, and isolator-raised germ-free mice models have been more or less successfully used for studying the interaction between *C. jejuni* and the human host. Numerous studies are hampered for several reasons; a detailed discussion, however, is beyond the scope of this review. In a nutshell, all mentioned models are expensive to construct and maintain, some are challenging in handling and availability, or lack of reproducibility [106]. Most murine models overcome these shortcomings, but suffer from sporadic colonization and/or absence of clinical disease manifestation [107]. Recently, combinations of various strategies have been used to construct gnotobiotic mice (GB), gnotobiotic humanized flora-associated mice (hfa), and gnotobiotic murine flora-associated mice (mfa) models [94]. The GB mice model was developed by treating 10- to 12-week old mice with a quintuple antibiotic regimen (consisting of ampicillin, vancomycin, ciprofloxacin, imipenem, and metronidazole) for six to eight weeks [108]. These gnotobiotic mice had been raised and housed under regular conditions and exhibited a fully developed immune system as opposed to isolator-raised germfree animals. Hfa mice and mfa mice were then generated by peroral recolonization of GB mice with complex human or murine microbiota, respectively. While mfa and conventional control mice expel the pathogen within a few days post infection, hfa mice are highly susceptible to *C. jejuni* infection and harbor the pathogen at high loads, further underlining that colonization resistance against (and susceptibility for) intestinal pathogens such as *C. jejuni* is due to the distinct host microbiota composition. Upon stable infection, GB and hfa mice display *C. jejuni* induced immunopathological features as seen in humans such as proinflammatory immune responses in the intestinal tract accompanied by significant apoptosis of the colonic epithelial cells, whereas severe clinical campylobacteriosis symptoms such as bloody diarrhea are lacking. In the following, Haag et al. showed that conventionally colonized infant mice infected with *C. jejuni* right after weaning at the age of three weeks developed self-limiting acute ulcerative enterocolitis like in “classical” human campylobacteriosis [109]. Interestingly, infant mice were susceptible to *C. jejuni* infection—again—due to relatively high commensal *E. coli* loads in their intestines by the age of three weeks, whereas over time *E. coli* burdens decreased subsequently rendering mice resistant to the pathogen [109]. Surprisingly, after the acute phase of infection, infant mice were asymptomatic carriers of *C. jejuni* over months. Strikingly, despite the absence of clinical sequelae, distinct inflammatory responses could be detected within the intestinal tract but also at extraintestinal tissue sites such as liver, kidney, and the lungs [109]. The infiltrating immune cells were mostly consisting of CD3+ T-lymphocytes, and to a lesser extent of B220+ B-lymphocytes. Thus, the infant *C. jejuni* infection model is not only mimicking “classical” self-limiting human campylobacteriosis, but might also be helping to unravel chronic (postinfectious) sequelae. Furthermore, gnotobiotic mice deficient in TLR-2, TLR-4, TLR-9, MyD88, and IL-10 genes have immensely contributed to our present understanding of the innate and adaptive immune responses and accompanying pro-inflammatory responses following *C. jejuni* infection [94, 96]. Remarkably, within six days upon *C. jejuni* infection, gnotobiotic IL-10-deficient mice developed severe ulcerative enterocolitis, which was not self-limiting and thus mimicking severe human campylobacteriosis like in immunocompromized (e.g., AIDS) patients [96]. Immunopathology was significantly less pronounced, however, when gnotobiotic IL-10−/− mice were additionally lacking the TLR-4 or, to a lesser extent, TLR-2 gene. Thus, signaling of *C. jejuni* lipooligosaccharide and lipoproteins play pivotal roles in the immunopathology of campylobacteriosis [94, 96].

## 8. Concluding Remarks

Although the exact species composition of the intestinal microbiota facilitating *C. jejuni* infection has not completely unraveled to date, the recent progress in establishing a plethora of reliable murine models for different degrees of *C. jejuni* infection such as the development of GB and hfa, gene-knockout, and weaned infant mice models are and will be useful to further understand the underlying molecular mechanisms of interactions between *C. jejuni* and the human intestinal microbiota as well as the human immune system. Ultimately, this could help to support the development of preventive or therapeutic strategies against *C. jejuni*-associated disease for instance through the establishment of appropriate diet schemes.

## Figures and Tables

**Table 1:** Summary of altered bacteria ratios in named noninfectious diseases.

Noninfectious disease	Names of bacteria altered	References
Type 1 diabetes	Decrease in *Lactobacillus* spp.	[43]

Type 2 diabetes	Increase in *Bacillus* spp. and *Lactobacillus* spp.	[44]

Obesity	Increase in *Bacteroidetes* Decrease (to low numbers) in *Bifidobacteria* spp.	[49, 50]

Inflammatory bowel disease (including Crohn's disease and ulcerative colitis)	Increase in *Enterobacteriaceae* Decrease in *Bacteroidetes* and certain *Firmicutes *	[46, 51]

Irritable bowel syndrome	Twofold increase in *Firmicutes* compared to *Bacteroidetes* with increase in *Clostridia* spp. and decrease in *Bifidobacteria* spp.	[47]

Celiac disease	Increase in *Lactobacillus* spp., *Bacteroides* spp., *Staphylococcus* spp., and *E. coli*. In some cases levels of *Bifidobacteria* spp. increase, while there is reduction in some cases. In children, there is increase in *Firmicutes* and low levels of *Bacteroidetes*.	[48, 52–54]

**Table 2:** Cytokines induced in *C. jejuni* infections.

Cytokines	Possible function	References
Chemokines: CC families: MIP-1*α*, MIP-1*β*, RANTES, and MCP-1	They act as chemoattractant agents for monocytes and T-lymphocytes.	[92]

Chemokines: CXC families: GRO-*α*, GRO-*γ*, IP-10, and MIG	GRO-*α* and GRO-*γ* attract neutrophils to sites of inflammation. IP-10 and MIG promote the chemotaxis of monocytes and activated T-lymphocytes.	[101]

Interleukins: IL-1B, IL-6, IL-8, IL-10, IL-12, IL-17A, IL-17F, IL-22, and IL-23	These chemoattractants and immune cells activators initiate Th1 and Th17 response. IL-5 and IL-6 activate STAT3. IL-22 maintains epithelial cell function and activation of macrophages.	[69, 93, 96, 100, 102, 103]

Interferon-gamma (IFN-*γ*)		[59, 69, 86, 93]

Tumor necrosis factor-alpha (TNF-*α*)	Plays a major role in production of IL-1 and IL-6	[86, 93]

Nuclear factor kappa-light-chain-enhancer (NF-*κ*B)signal transducer and activator of transcription 3 (STAT3)	Stimulates production of chemokines limits host inflammatory response in the gut	[86, 92, 104, 105]
